# Empowering health care consumers & understanding patients' perspectives on AI integration in oncology and surgery: A perspective

**DOI:** 10.1002/hsr2.2268

**Published:** 2024-07-23

**Authors:** Wireko Andrew Awuah, Nicholas Aderinto, Jeisun Poornaselvan, Joecelyn Kirani Tan, Muhammad Hamza Shah, Patrick Ashinze, Anushka G. Pujari, Hareesha Rishab Bharadwaj, Toufik Abdul‐Rahman, Oday Atallah

**Affiliations:** ^1^ Faculty of Medicine Sumy State University Sumy Ukraine; ^2^ Internal Medicine Department LAUTECH Teaching Hospital Ogbomoso Nigeria; ^3^ School of Medicine University College Dublin Dublin Ireland; ^4^ Faculty of Medicine University of St Andrews St. Andrews Scotland UK; ^5^ School of Medicine Queen's University Belfast Belfast UK; ^6^ Faculty of Clinical Sciences University of Ilorin Ilorin Nigeria; ^7^ Royal College of Surgeons in Ireland Dublin Ireland; ^8^ Faculty of Biology, Medicineand Health The University of Manchester Manchester UK; ^9^ Department of Neurosurgery Hannover Medical School Hannover Germany

**Keywords:** artificial intelligence, oncology, patients perspectives, surgery

## Abstract

**Introduction:**

Artificial intelligence (AI) is transforming oncology and surgery by improving diagnostics, personalizing treatments, and enhancing surgical precision. Patients appreciate AI for its potential to provide accurate prognoses and tailored therapies. However, AI's implementation raises ethical concerns, data privacy issues, and the need for transparent communication between patients and health care providers. This study aims to understand patients' perspectives on AI integration in oncology and surgery to foster a balanced and patient‐centered approach.

**Methods:**

The study utilized a comprehensive literature review and analysis of existing research on AI applications in oncology and surgery. The focus was on examining patient perceptions, ethical considerations, and the potential benefits and risks associated with AI integration. Data was collected from peer‐reviewed journals, conference proceedings, and expert opinions to provide a broad understanding of the topic. The perspectives of patients was also emphasized to highlight the nuances of their acceptance and concerns regarding AI in their health care.

**Results:**

Patients generally perceive AI in oncology and surgery as beneficial, appreciating its potential for more accurate diagnoses, personalized treatment plans, and improved surgical outcomes. They particularly value AI's role in providing timely and precise diagnostics, which can lead to better prognoses and reduced anxiety. However, concerns about data privacy, ethical implications, and the reliability of AI systems were prevalent. Consequently, trust in AI and health care providers was deemed as a crucial factor for patient acceptance. Additionally, the need for transparent communication and ethical safeguards was also highlighted to address these concerns effectively.

**Conclusion:**

The integration of AI in oncology and surgeryholds significant promise for enhancing patient care and outcomes. Patients view AI as a valuable tool that can provide accurate prognoses and personalized treatments. However, addressing ethical concerns, ensuring data privacy, and building trust through transparent communication are essential for successful AI integration. Future initiatives should focus on refining AI algorithms, establishing robust ethical guidelines, and enhancing patient education to harmonize technological advancements with patient‐centered care principles.

## Highlights

- Artificial intelligence (AI) has ushered in transformative potential in surgery and oncology, including enhanced patient care through novel treatment plans and diagnosis, and improved precision.
- Although AI offers numerous benefits in the field of surgical oncology, it also presents several challenges, including ethical issues, safety and privacy risks, bias in algorithms, and increased health disparities due to limited access.
- As important as recognizing and addressing the various viewpoints of patients and their families on AI usage are, these patients' perspectives on AI in surgery and oncology have been vastly underexplored.
- Future initiatives should prioritize AI algorithm refinement, ethical guidelines, and robust data governance mechanisms because successful AI integration into health care necessitates a balance between technological advancement, the preservation of fundamental ethical principles, and patient‐centered care.

## 1 INTRODUCTION

Artificial intelligence (AI) has introduced transformative possibilities in oncology and surgery, including improved diagnostics, personalized treatment strategies, and enhanced precision. Patients regard AI as a critical ally, especially in cancer care, for it offers more accurate prognoses and tailored treatments.^1^ Additionally, the timely diagnosis facilitated by AI's data‐driven algorithms is highly valued.

In oncology, AI's application spans diagnosis, treatment planning, and predicting treatment outcomes, potentially increasing the accuracy and efficiency of cancer care.^2^ Algorithms excel in processing complex medical images, pinpointing minute tumor changes that might elude the human eye.^3^ In the surgical sphere, AI‐enhanced robotic systems provide surgeons with greater precision and control, leadingto minimallyinvasive procedures, shorter hospital stays, and quicker recovery times. AI also plays a vital role in preoperative planning, intraoperative guidance, and postoperative monitoring.^4^

Despite the revolutionary potential of AI in surgery and oncology, its deployment comes with significant challenges and considerations.^3^ The development of AI algorithms often requires large datasets, necessitating collaboration and data sharing among health care institutions.^5^ Ensuring the safety and reliability of AI systems is paramount, as errors or biases in algorithms could lead to dire consequences for patients. Resistance from health care professionals might emerge, fueled by concerns over AI replacing their roles or diminishing their decision‐making autonomy. Ethical issues, such as privacy concerns and the potential for biases within AI algorithms, represent substantial hurdles. Additionally, the impact of AI on health disparities warrants attention, as access to AI technologies may vary across different demographics.^6^

The rapid proliferation of publications in this field underscores an increasing awareness of the importance of understanding and integrating the perspectives of both patients and physicians regarding AI in oncology and surgical care. From the physician's standpoint, AI is seen as a tool to augment their capabilities, yet there are apprehensions about skill atrophy and loss of professional autonomy.^5^ Trust in AI's recommendations is another area of concern. Conversely, patients perceive AI as a double‐edged sword; while it can provide more accurate diagnoses and advanced treatments, there is also fear regarding the delegation of critical health decisions to machines.^5^, ^6^

While AI in medicine aims to improve patient care, the emotional and psychological impacts of such technologies on patients have not been adequately addressed.^4^, ^6^ A seamless integration of AI into health care necessitates not only technological innovation but also a profound understanding of human needs and concerns.

## 2 PATIENTS' PERSPECTIVES ON AI IN ONCOLOGY AND SURGERY

The convergence of AI and oncology heralds a significant transformation in cancer care, offering enhanced diagnostics, personalized treatment plans, and improved outcomes for patients.^7^ This integration is viewed with optimism by patients and their families, who regard AI as a pivotal support in their battle against cancer. Tools like predictive analytics and genomic profiling are at the forefront, providing more accurate prognoses and tailored treatment recommendations, thus boosting patient confidence in the health care decisions made by oncologists^1^, ^8^ AI's ability to rapidlyprocess vast datasets facilitates the creation of highly personalized treatment plans, aligning closely with individual genetic and clinical profiles, thereby not only potentially reducing side effects but also actively engaging patients in their treatment process^9^, ^10^ Furthermore, the importance of timely diagnosis in cancer treatment is magnified through AI's role in early detection, utilizing image analysis and data‐driven algorithms to achieve faster and more accurate diagnoses, reducing anxiety and enabling critical, timely interventions.^10^, ^11^

However, this optimism for AI's potential in cancer care must be balanced with a careful consideration of its ethical implications, particularly concerning the collection, storage, and usage of sensitive health data. The challenges unique to AI, such as potential biases and inequities, necessitate a more sophisticated approach to data protection, distinguishing it from other digital health advancements like Electronic Health Records. These concerns are practical, not just theoretical, affecting patient outcomes and the integration of AI technologies into health care systems.

The integration of AI into surgical practices ushers in a new era, enhancing surgical precision and recovery times. Patient perceptions of AIin surgeryare crucial, reflecting their hopes and concerns regarding surgical interventions. AI technologies, including robotic‐assisted surgery systems, provide surgeons with unparalleled precision, minimizing tissue damage, reducing scarring, and accelerating recovery—a set of improvements highly valued by patients^12^, ^13^, ^14^ One of the key benefits for patients is the potential for shorter recovery periods, with AI assisting in the development of personalized postoperative care plans based on predictive analytics.^15^ This not only ensures a quicker return to daily activities but also reduces postsurgical discomfort. Additionally, the capability of AI to analyze extensive patient data for early detection of potential complications adds an extra layer of safety to the surgical process.^16^

Nonetheless, establishing and maintaining trust in AI systems and surgical teams is essential, as patient perspectives frequently highlight the importance of trust in the adoption of AI into their surgical care. Transparencyin explaininghow AI systems aid in surgical decision‐making, coupled with comprehensive education on the role, benefits, and limitations of AI in surgery, is crucial for building this trust.^17^ Providing patients with clear, understandable information empowers them to make informed decisions confidently, underscoring the pivotal role of education in patient perceptions of AI in surgery.

## 3 DISCUSSIONS AND PROSPECTS

### 3.1 From data to decision: Clinicians as interpreters of AI in health care

In AI applications within oncology and surgery, physicians and surgeons are crucial in bridging the advanced technologies to patient perspectives. They are instrumental in interpretingthe outputs from AI algorithms in these fields,^18^ translating complex AI‐generated data into terms understandable to patients. This role is critical, as effective communication ensures patients are aware of both the benefits and limitations of AI in their treatment. Addressing patient concerns about AI's role in cancer treatment or surgical procedures is essential for building trust and understanding. Moreover, while AI provides valuable insights and recommendations, the ultimate decisions about treatment options rest with the patients and their health care providers.^19^ Physicians and surgeons engage patients in shared decision‐making, taking into account their preferences, values, and medical histories. This collaborative approach fosters patient trust and satisfaction with AI‐integrated health care.

Beyond interpretation and recommendation, clinicians have a critical role in continuously monitoring AI systems' performance in clinical practice. They collect feedback from patients on their experiences with AI‐assisted care, which is vital for refining AI algorithms and ensuring they meet patients' evolving needs and expectations. This feedback loop helps maintain the alignment of AI technologies with patient care objectives in oncology and surgery.

### 3.2 Empowering care through AI and bridging the patient knowledge gap

To enhance patient understanding and bridge knowledge gaps about AI in health care, integrating patients into decision support systems is essential. These systems can significantly empower oncologists and surgeons to provide personalized care.^20^ By incorporating AI tools into clinical workflows, health care professionals can reduce administrative tasks, allowing for more meaningful interactions with patients.^21^ Continuous education and training for health care providers are also crucial as AI technology advances.

The integration of AI into oncology and surgery needs to align with the four core principles of medical ethics: autonomy, informed consent, data protection, and empathy.^22^ Policymakers and regulatory bodies play a key role in establishing an ethical and legal framework for AI use that respects patient rights.^23^ This framework should address patient privacy, data security, and the transparency of algorithms to ensure ethical AI application in health care.

A significant concern with AI in health care is the risk of cyberattacks, which have become more prevalent with the introduction of new data variables through AI integration. Many of these cyberattacks are detected only after a data breach has occurred.^24^ Therefore, health care administrative bodies must focus on mitigating these risks to build patient confidence in new technologies. Accountability in AI integration is paramount and should be managed within an established framework. Implementing strong data governance and sharing protocols is necessary to support AI‐driven research while protecting patient information and building trust.

Patient advocacy organizations should lead in promoting patient‐centered care in oncology and surgery, including in research activities. Their role in educating patients about AI and facilitating dialogue between patients and health care providers is vital for empowering patients to make informed decisions. Such efforts ensure that AI applications align with patient preferences and expectations. Encouraging extensive patient involvement in research, clinical trials, and other innovative programs is also important for advancing patient‐centered health care.

### 3.3 Integrating radiogenomics

Radiogenomics, an emerging field at the intersection of radiology and genomics, leverages the power of advanced imaging (radiomics) and genetic data (genomics) to provide a comprehensive view of a tumor's characteristics. In the context of AI integration in oncology and surgery, particularlywithin urology, radiogenomics signifies a paradigm shift in how cancers, such as those of the kidney, bladder, and prostate, are approached. For instance, in renal cancer management, radiogenomics aids in distinguishing between benign and malignant lesions, thus informing surgical decisions and potentially sparing patients from unnecessary treatments.^25^ This precision is achieved by analyzing the radiomic features from imaging alongside genomic data, which together predict the aggressiveness of the tumor and guide the choice of surgical intervention.

Similarly, for prostate cancer, combining AI with radiogenomics has been instrumental in assessing tumor aggressiveness. This approach supports clinicians in deciding between conservative management and more radical surgical options, tailoring treatments to the individual patient's needs and minimising the risk of overtreatment.^26^ In fact, the integration of AI and radiogenomics enhances the accuracy of prostate biopsies by ensuring that only clinically significant areas are targeted.^27^ This reduces the need for repeated biopsies and improves the detection of significant cancers, thus directly impacting the patient's treatment pathway and quality of life post‐surgery.^28^ Successful implementation of AI and radiogenomics in surgical planning and oncology treatment not only promises a future where treatments are highly personalized but also emphasizes the importance of patient and family understanding and involvement in the care process. By providing a clearer understanding of complex procedures and what to expect, AI and radiogenomics reduce patient anxiety and build trust in the health care team.

## 4 CONCLUSION

Integrating AI in oncology and surgery offers remarkable potential for improving patient care, diagnostics, and treatment outcomes. Patients and their families perceive AI as a source of hope, appreciating its ability to provide a more accurate prognosis, personalized treatment recommendations, and timely diagnosis. In surgery, patients highly value the enhanced precision, reduced recovery times, and minimize risks that AI offers. However, ethical concerns, particularly regarding data privacy and trust in health care systems, demand clear explanations and trust‐building measures from clinicians. Future initiatives should prioritize refining AI algorithms, ethical guidelines, and robust data governance mechanisms. Ultimately, the successful integration of AI into health care necessitates a balance between technological advancement, the preservation of fundamental ethical principles, and patient‐centered care.

## AUTHOR CONTRIBUTIONS

The study was conceptualized and supervised by Wireko Andrew Awuah and Jeisun Poornaselvan Material preparation, data collection, and analysis were performed by all authors. The first and final draft of the manuscript was written by all authors. All authors read and approved the final manuscript.

## CONFLICT OF INTEREST STATEMENT

The authors declare no conflict of interest.

## TRANSPARENCY STATEMENT

The lead author Wireko Andrew Awuah affirms that this manuscript is an honest, accurate, and transparent account of the study being reported; that no important aspects of the study have been omitted; and that any discrepancies from the study as planned (and, if relevant, registered) have been explained.

## Data Availability

Data availabilityis not applicable to this article as no new data were created or analyzed in this study.
